# Recent Trends in Health Literacy Research, Health Status of the Population and Disease Prevention: An Editorial

**DOI:** 10.3390/ijerph19148436

**Published:** 2022-07-10

**Authors:** Agnieszka Barańska, Anna Kłak

**Affiliations:** 1 Department of Medical Informatics and Statistics with e-Health Lab, Medical University of Lublin, K. Jaczewskiego 5 Street, 20-059 Lublin, Poland; agnieszkabaranska@umlub.pl; 2 Department of Environmental Hazards Prevention, Allergology and Immunology, Medical University of Warsaw, Banacha 1a Street, 02-091 Warsaw, Poland

One challenge for the development of healthcare systems worldwide is to shape society’s health literacy. The World Health Organisation (WHO) defines health literacy as the cognitive and social skills that determine the motivation and ability of individuals to access, understand, and use information in ways which promote and maintain good health [1,2]. Health literacy improves people’s ability to better understand health-related notions and may increase their capacity to take responsibility for their health, which is of great importance in prophylaxis [3]. Health literacy is not just the result of an individual’s capabilities, but also of the demands and complexities of the healthcare system [4]. The shaping of health literacy is highly influenced by the healthcare systems in a given country, as well as by individual and environmental factors present in the interactions between individuals and the systemic demands of the healthcare system [5].

Health literacy may impact patient adherence, the frequency of using medical services, and the number of hospitalisations [6]. Studies have confirmed this thesis, and have indicated a correlation between low health literacy, poor health, and the unjustified frequent use of health services; this is particularly the case for admission rooms and more frequent hospitalisations [7,8,9,10], which create a higher financial burden for the healthcare system. Nearly half of the European population have low health literacy [1], which can also be observed in the United States [11,12].

Health-literacy-related literature shows a range of dependencies and reasons for low health literacy in families [13,14], which may influence: parents/carers when providing medication dosage to children, breastfeeding time, and child health outcomes, such as the symptoms of depression, asthma, etc. [15,16,17,18]. One basic tool for shaping health literacy is education. Sparse health knowledge is associated with more frequent emergency assistance, less frequent use of prophylactic services, a lower capacity to interpret health labels and notices, higher mortality, and higher healthcare expenses [14]. Low health literacy may also be a barrier to taking care of chronically ill patients, and may interfere with taking measures at the central level regarding the functioning of healthcare systems, with the objectives of prophylaxis, early diagnosis, and treatment [19,20].

Studies on health literacy are becoming increasingly important in the context of public health and health promotion. Human behaviour is becoming an integral factor that determines health maintenance and disease development [21]. The essence of a holistic approach to health lies in acknowledging the complex relationships between a person and their environment, as well as in highlighting the dynamic, interactive, and multidimensional nature of health [22]. Health literacy is a determinant of health behaviour and of preventing disease. It must be noted that health literacy is associated with the quality of decisions related to healthcare, infectious and chronic disease prevention, and health promotion, to maintain or improve quality of life [19,20,21,22].

Health literacy is also the knowledge of health behaviour and of the prevention and elimination of diseases, major threats, and harmful factors for health. Health behaviour has been considered the most significant element of health promotion and disease prevention and needs to be developed, especially in primary healthcare [23]. Health behaviour is defined as any intentional activity of an individual aimed at healthcare, regardless of its efficiency [24]. This issue has been the subject of theoretical deliberation and studies in different fields. The results lead to the assertion that non-compliance with the guidelines for a healthy lifestyle (any detrimental health behaviour) is a risk factor for diseases in civilisation [25,26,27,28]. Lifestyle is of great importance for health. The recommendations indicating the foundations of modern healthcare emphasise the necessity to guarantee a structure that facilitates the implementation and evaluation of health promotion and the prevention of infectious and chronic diseases, simultaneously monitoring those health behaviours that delay these diseases and complications.

It is extremely important to carry out scientific research that analyses the relationships between health awareness, health behaviours, and the health status of the population. A Special Issue on “Recent Trends in Health Literacy Research, Health Status of the Population and Disease Prevention” (IJERPH) presents publications reviewing health literacy, the capacities and behaviours of the population that impact their health, and the prevention of chronic and infectious diseases. The studies evaluate the health capacities and behaviours of the population, attitudes toward preventative actions in healthcare (e.g., immunisation and healthy lifestyle), and systemic factors, policies and practices that enhance or inhibit the health of the population. The results of these studies (as well as infodemiological studies) evaluating the variables impacting the health of the population are presented. Reports on methods for assessing the health literacy of individuals and the population are crucial, and ensure the review of up-to-date national and international political initiatives in health literacy.
